# Hydrogels promote periodontal regeneration

**DOI:** 10.3389/fbioe.2024.1411494

**Published:** 2024-05-17

**Authors:** Huiying Sun, Jiayi Luan, Shujun Dong

**Affiliations:** ^1^ The First Outpatient Department, Jilin Provincial Key Laboratory of Tooth Development and Bone Remodeling, School and Hospital of Stomatology, Jilin University, Changchun, China; ^2^ Foshan Stomatology Hospital and School of Medicine, Foshan, Guangdong, China

**Keywords:** hydrogel, periodontitis, periodontal regeneration, guide tissue regeneration, cell transplantation, scaffolds, drug delivery systems, tissue engineering

## Abstract

Periodontal defects involve the damage and loss of periodontal tissue, primarily caused by periodontitis. This inflammatory disease, resulting from various factors, can lead to irreversible harm to the tissues supporting the teeth if not treated effectively, potentially resulting in tooth loss or loosening. Such outcomes significantly impact a patient’s facial appearance and their ability to eat and speak. Current clinical treatments for periodontitis, including surgery, root planing, and various types of curettage, as well as local antibiotic injections, aim to mitigate symptoms and halt disease progression. However, these methods fall short of fully restoring the original structure and functionality of the affected tissue, due to the complex and deep structure of periodontal pockets and the intricate nature of the supporting tissue. To overcome these limitations, numerous biomaterials have been explored for periodontal tissue regeneration, with hydrogels being particularly noteworthy. Hydrogels are favored in research for their exceptional absorption capacity, biodegradability, and tunable mechanical properties. They have shown promise as barrier membranes, scaffolds, carriers for cell transplantation and drug delivery systems in periodontal regeneration therapy. The review concludes by discussing the ongoing challenges and future prospects for hydrogel applications in periodontal treatment.

## Graphical Abstract

**Figure:**
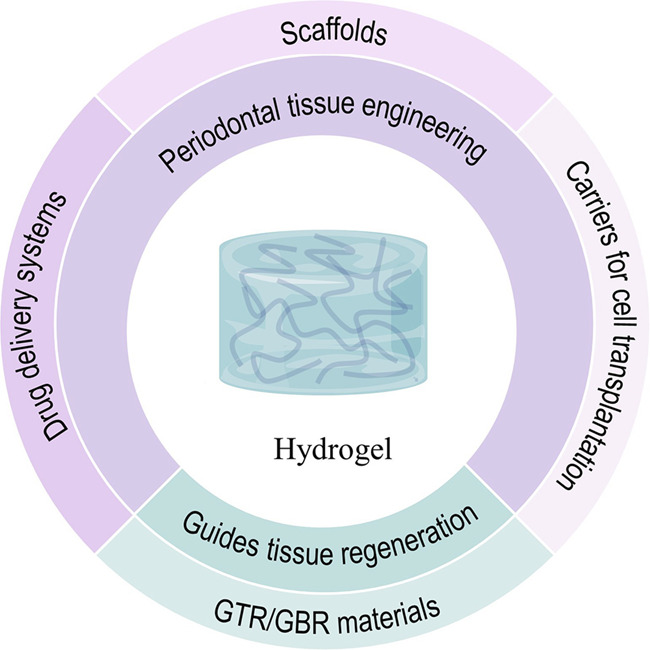
Functional hydrogel for periodontal regeneration therapy.

## 1 Introduction

Periodontitis, a chronic condition leading to the inflammatory destruction of periodontal supporting tissues, is a primary cause of tooth loss in adults. With an estimated 1.1 billion people worldwide suffering from severe periodontitis, the disease significantly compromises chewing efficiency and causes eating difficulties. Additionally, the cosmetic effects of periodontitis, such as changes to the jawline and appearance when smiling due to receding gums and loose teeth, can adversely affect social interactions and the patient’s quality of life (Kononen et al., 2019; Wen et al., 2021). Despite current clinical therapies for periodontitis, including surgical interventions, root planing, and various scaling techniques, along with local antibiotic injections, aiming to alleviate symptoms and manage the disease progression (Deas et al., 2016; Slots, 2017; Kwon et al., 2021; Wadia, 2021), a full restoration of periodontal tissue function and structure remains unachieved. Consequently, there is an ongoing search for innovative therapeutic strategies, such as guided tissue regeneration (GTR)/guided bone regeneration (GBR), bone grafts, growth factors, stem cell therapy, and periodontal tissue engineering, to repair the structure and function of damaged periodontal tissues. While there have been advancements, challenges remain in enhancing the success and predictability of these treatments (Sam and Pillai, 2014; Giannobile and McClain, 2015; Hu et al., 2018), highlighting the demand for efficient and safe periodontal regeneration methods.

Strategies for periodontal regeneration are categorized into GTR and periodontal tissue engineering. The concept of GTR was introduced by Nyman et al. in 1982 (Gottlow et al., 1990), emphasizing the use of membranes to prevent gingival epithelium from expanding toward the tooth root, thereby encouraging osteoblasts and periodontal ligament stem cells (PDLSCs) to form new attachments (Gottlow et al., 1990). The selection and application of barrier membranes are crucial for the effectiveness of GTR technology, which offers both absorbable and non-absorbable varieties (Alqahtani, 2023; Zhu et al., 2023). Non-absorbable membranes, such as titanium and polytetrafluoroethylene, are known for their excellent mechanical and biocompatibility properties, suitable for independent usage in clinical settings (Mizraji et al., 2023). However, their non-resorbable nature necessitates a second surgical procedure for removal, increasing the risk of infection and potentially damaging newly formed periodontal tissue. In contrast, absorbable membranes, made from synthetic or natural polymers like polylactic acid, eliminate the need for a second surgery as they are absorbed by the body during the healing process. These membranes are typically cell-affine, facilitating cell attachment and proliferation, and are easy to handle. However, absorbable membranes possess relatively low mechanical strength and a degradation rate that is difficult to control precisely, potentially limiting their effectiveness in periodontal regeneration (Sasaki et al., 2021). The success of GTR technology in periodontal regeneration, despite its widespread application, depends on several factors including the specific tooth anatomy, the precision of the surgical procedure, postoperative care, and proper indication selection (Villar and Cochran, 2010). Therefore, for enhanced effectiveness and therapeutic outcomes of periodontal regeneration, physicians implementing GTR treatments must consider these variables.

Tissue engineering for periodontal regeneration is an interdisciplinary field that merges clinical medicine, biology, and materials science (Liu et al., 2018; Liang et al., 2020), aiming to rebuild damaged periodontal tissues through the use of stem cells, growth factors, and scaffold materials (Han et al., 2014). A typical approach involves isolating and culturing specific cells *in vitro*, seeding them onto a scaffold that mimics the target tissue’s natural extracellular matrix (ECM), and then implanting the assembly into the patient’s target site. Stem cells are crucial for periodontal tissue engineering due to their self-renewal capability and potential to differentiate into various cell types (Iwasaki et al., 2022). Stem cells for periodontal tissue are often derived from dental pulp, bone marrow mesenchymal stem cells, and PDLSCs (Gao et al., 2024), which can differentiate into specific periodontal cells such as osteoblasts and fibroblasts, aiding in tissue regeneration. Growth factors play a vital role in regulating cell behavior and promoting cell migration, proliferation, differentiation, and neovascularization in periodontal tissue regeneration (Kale et al., 2022). Scaffold materials serve as the foundation for tissue engineering, offering a structure for cell attachment, growth, and differentiation. Current periodontal scaffolds are made from synthetic materials like polycaprolactone (PCL) and polylactic acid-glycolic acid copolymer (PLGA), as well as natural materials like collagen (Daghrery et al., 2023; Ostrovidov et al., 2023). Despite significant progress, periodontal tissue engineering faces challenges, including the need to improve the success rate of tissue engineering repairs, as outcomes are often unpredictable, and ensuring the stability of *in vivo* applications (Galli et al., 2021). The complexity of periodontal tissue, comprising intricate tissue architectures and diverse types, means current treatments cannot fully restore the tissue’s original structure and function. Thus, periodontal regeneration remains a complex and challenging domain, requiring ongoing scientific research and technological advancements to address existing challenges (de Jong et al., 2017).

Hydrogels have shown considerable promise in clinical periodontal tissue regeneration, offering a biomimetic environment similar to natural biological tissues and the ECM (Sánchez-Cid et al., 2022). These polymeric materials can absorb and retain large amounts of water while maintaining a solid structure, thanks to a three-dimensional network of polymer chains interconnected through physical or chemical cross-links. Their high water content endows hydrogels with softness and biocompatibility, fostering an ideal setting for cell adhesion, proliferation, differentiation, and growth (Radulescu et al., 2022). The physicochemical properties of hydrogels can be customized by adjusting their functional elements, cross-linking methods, and synthetic ingredients, allowing them to meet the specific mechanical and biological requirements of different tissues. Hydrogels have found applications in drug delivery, regenerative medicine (Dimatteo et al., 2018; Mantha et al., 2019; Tavakoli and Klar, 2020) and more, thanks to their ability to be tailored for specific medical uses (Jacob et al., 2021). The primary ingredients of natural hydrogels are polysaccharides and proteins, such as gelatin, hyaluronic acid, and chitosan (CS). These substances are favored for their natural origins. However, their low mechanical strength and limited stability render them less suitable for clinical applications, despite their proven biosafety and biodegradability in both animal and clinical trials (Catoira et al., 2019). By contrast, synthetic hydrogels are formed through physical or chemical crosslinking and include polymers like polyvinyl alcohol. These hydrogels offer superior mechanical properties and consistent mass stability, but the presence of unreacted monomers, initiators, or crosslinkers can cause inflammation or cytotoxicity (Branco et al., 2022). To enhance the qualities of composites, hydrogel can also be used in combination with other materials (Wang Y. et al., 2020; Patel and Koh, 2020).

**SCHEME 1:**
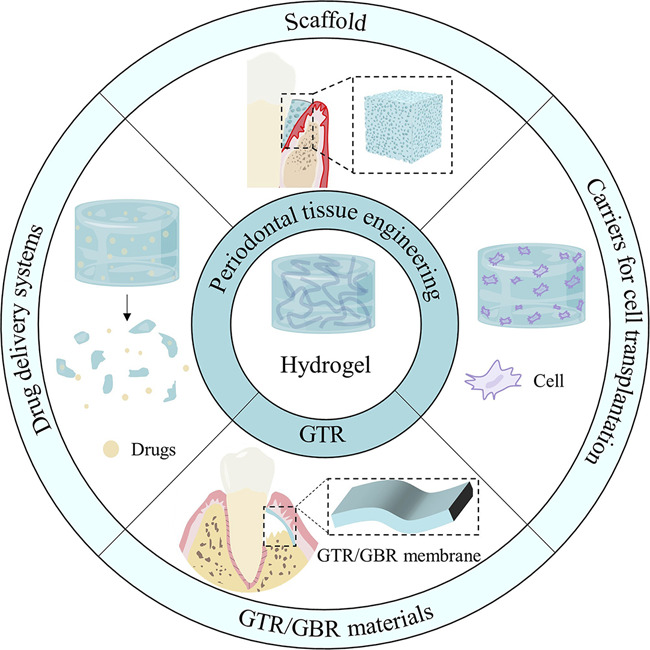
Hydrogels promote periodontal regeneration.

This study describes the use of hydrogels in periodontal tissue engineering, focusing on their roles as GTR barrier membranes, scaffolds, carriers for cell transplantation and drug delivery systems (Scheme 1). It highlights the research progress of hydrogels in different application directions, and the potential for further development of hydrogel materials to aid in the regeneration of periodontal tissues.

## 2 Hydrogels as guided tissue regeneration/guided bone regeneration materials

In GTR and GBR, the membrane plays a crucial role in the regeneration of periodontal tissue. An ideal barrier membrane should possess several characteristics (Gao et al., 2022; Mirzaeei et al., 2022): First, it must be biocompatible with the surrounding periodontal tissue to minimize immune responses and inflammation. Second, it should have adequate mechanical strength and flexibility to maintain its shape and provide necessary space for new tissue growth during surgery. Furthermore, the membrane should feature moderate porosity to prevent epithelial cell invasion into the bone defect area while allowing the exchange of growth stimuli and nutrients. Biodegradability is another important factor, requiring the membrane to degrade at a rate compatible with periodontal tissue regeneration. Lastly, the material should be easy to handle clinically, including cutting, placing, and securing. Recent research has focused on improving the biomechanical properties of GTR/GBR membranes and introducing new therapeutic functionalities, such as osteoinduction or antibacterial activity (Gao et al., 2022; Mirzaeei et al., 2022). Due to their excellent biosafety and adjustable properties, hydrogels are considered as potential materials for GTR/GBR membranes (Alauddin et al., 2022). For instance, a study by Humber et al. (2010) demonstrated the effectiveness of an *in situ* formed polyethylene glycol hydrogel membrane as a GBR barrier in supporting bone tissue repair. However, the use of hydrogels as GTR/GBR membranes faces challenges, particularly in mechanical performance, often failing to meet clinical requirements. Therefore, ongoing research aims to enhance the biomechanical properties of hydrogels through formula optimization, cross-linking density adjustment, and the inclusion of functional additives to broaden their therapeutic applications.

Enhancing the biomechanical characteristics of hydrogels can be achieved through the incorporation of inorganic fillers (Bee and Hamid, 2022). Commonly used inorganic materials in periodontal tissue engineering include inorganic phosphate ceramics such as hydroxyapatite (HA) and β-tricalcium phosphate. Akansha Kishen et al. (2023) introduced a novel hydroxyapatite-reinforced polymer hydrogel membrane by combining hydroxyapatite with alginate, a naturally occurring polymer known for its excellent biological compatibility and biodegradable properties, capable of forming a gel structure in the body (Zhang et al., 2021). Given hydroxyapatite’s osteoinductive qualities, it enhanced the hydrogel membrane’s mechanical strength and accelerated periodontal tissue regeneration, suggesting its potential in periodontal regeneration therapy as a GTR membrane. The asymmetric barrier membrane developed by He et al. (2021) consisted of agarose, hydroxyapatite carbonate (CHA), and ε-polylysine. CHA is derived from HA through chemical modification, maintaining HA’s high mechanical strength and biocompatibility while enhancing bone conductivity. This enhancement supports bone tissue development and regeneration (Wang et al., 2024). Increasing the CHA concentration in the hydrogel significantly raised the storage modulus of the barrier membrane. Specifically, a CHA content of 10% resulted in a 30 kPa increase in storage modulus compared to pure agarose gel, indicating that adjusting the CHA content can enhance the membrane’s mechanical properties and its barrier effectiveness. Moreover, membranes with higher CHA concentrations showed increased mineralization capacity and osteogenesis-promoting effects. The incorporation of ε-polylysine provided antimicrobial properties, useful in preventing or reducing periodontitis recurrence. Thus, this barrier membrane offers improved mechanical properties, bone tissue regeneration promotion, and antimicrobial defense, contributing significantly to GTR for periodontal tissue regeneration and rebuilding.

The addition of graphite and its derivatives into hydrogels notably enhances their mechanical properties. Graphene, the primary conductive carbon-based substance, is recognized for its excellent biological activity and bone repair effects (Sattar, 2019). It is one of the strongest materials known, exhibiting remarkable toughness, biocompatibility, stability, thermal conductivity, and a large specific surface area. These features make graphene widely used in biosensing, bioimaging, and tissue regeneration. The structural and mechanical properties of hydrogels, such as toughness, tear strength, and elastic modulus, can be influenced by graphene through mechanisms such as hydrogen bonding, electrostatic interactions, and hydrophobicity (Zare et al., 2024). Lu et al. (2016) described a graphene hydrogel (MGH) membrane consisting of multilayered chemically modified graphene sheets, notable for its strength and flexibility. The membrane’s average tensile modulus was comparable to that of rat skull tissue, ensuring strong adherence to defects in rat skull models and stability post-transplantation. Microcomputed tomography (CT) images verified new bone tissue development beneath the MGH membrane after 8 weeks, indicating its role in physical support and osteoblast growth promotion, thereby facilitating bone tissue regeneration. Consequently, MGH membranes present a novel approach for the clinical management of bone defects as GBR membranes.

Fibers are materials made up of continuous or discontinuous filaments that possess significant stiffness. However, they face challenges in forming three-dimensional (3D) structures. To overcome this, numerous studies have focused on incorporating them into hydrogels to create composite materials that leverage the strengths of both components. Fiber-reinforced hydrogels, for example, are enhanced by the addition of fibers, making their structure more akin to the ECM, thereby promoting better cellular functions (Khorshidi and Karkhaneh, 2018). ε-caprolactone is converted into PCL, a polymer with a low melting point, through ring-opening polymerization (Hernandez et al., 2017). This material is biocompatible and versatile, thanks to 3D printing technologies. Dubey et al. (2020) developed a highly porous PCL network via melt electrowriting, which was then integrated into a gelatin methacryloyl (GelMA) hydrogel supported by amorphous magnesium phosphate. This integration significantly improved the hydrogel’s mechanical properties, enabling it to withstand nearly 70 kPa of stress under 20% deformation. This enhancement not only helped the hydrogel maintain its structure under stress but also acted as an effective barrier against epithelial cell invasion. The fibrous network structure of the hydrogel facilitated a faster rate of mineralization when used as a GBR membrane, supporting bone tissue formation more effectively. This enhancement was attributed to the rigid support of the PCL network, which also slowed down degradation, thereby extending its lifespan *in vivo* and providing sustained support for bone tissue regeneration. Overall, this bioactive, fiber-reinforced hydrogel membrane presents significant potential for GBR applications.

A double network (DN) hydrogel comprises two interpenetrating polymer networks, characterized by a loose network structure and a polyelectrolyte network structure with a high cross-linking density. This configuration imparts unique structural characteristics to the DN hydrogel (Zhou et al., 2020; Li S. et al., 2021; Hanyková et al., 2023), including a stable support from the cross-linked neutral network, which also absorbs external stress through the polyelectrolyte network structure. Besides maintaining the physical properties of conventional hydrogels, the dual network significantly improves toughness and mechanical properties (Ning et al., 2022), offering a synergistic performance that enhances mechanical strength and inhibits phase separation (Huang et al., 2023). Chichiricco et al. (2018) developed a photochemically cross-linked hydrogel membrane by combining a silylated hydroxypropyl methylcellulose network with a methacrylated carboxymethyl CS network, achieving rapid gelation. The presence of the second polymer reduced gelation time significantly, forming a hydrogel membrane within 2 min of irradiation. This rapid curing process, combined with the membrane’s adaptability to wound shapes, enhances the cell barrier effect, making it easier to use during surgery. Furthermore, the double network structure provided a synergistic increase in mechanical strength, evidenced by a higher Young’s modulus compared to single-network hydrogels. This enhancement in mechanical properties, attributed to the supportive interaction between the two networks, makes the hydrogel membrane particularly valuable for applications requiring robust barrier membranes, such as in periodontal regeneration therapy.

Bacterial infections are a prevalent complication with medical implants. Additionally, microbes cause periodontitis, a serious gum disease. The site of periodontal tissue healing often becomes contaminated, which hampers effective periodontal regeneration. Therefore, ensuring the antibacterial efficacy of GTR/GBR barrier membranes is crucial. Incorporating antibacterial substances into these membranes helps control bacterial colonization and reduces infection risk during the initial healing stages. The most common antibacterial agents used in GTR/GBR are antibiotics, such as minocycline, doxycycline, metronidazole, and vancomycin (Toledano-Osorio et al., 2022). A study introduced the broad-spectrum antibiotic chloramphenicol and the local anesthetic lidocaine into a bioresorbable hydrogel membrane, creating a drug-eluting system with potential for periodontal GTR (Vasconcelos et al., 2022). However, excessive reliance on antibiotics can lead to increased multidrug resistance and reduced treatment effectiveness. To overcome these challenges, alternative strategies are necessary to complement or replace traditional antibiotic approaches. Natural materials have been used since ancient times in the antibacterial field to enhance therapeutic efficacy and reduce bacterial resistance. These can be utilized either alone or alongside conventional antibiotics (Malczak and Gajda, 2023). Hinokitiol, a natural compound from monoterpenoids with antibacterial properties, inhibits bacterial cell respiration, DNA, and protein synthesis (Chang et al., 2019). By using N-(3-dimethylaminopropyl)-N′-ethylcarbodiimide hydrochloride to cross-link gelatin and hyaluronic acid, and incorporating hinokitiol, Chang et al. (2021) developed a hydrogel-regenerated membrane. This membrane, laden with hinokitiol, showed potent antibacterial activity against both *Staphylococcus aureus* and *Escherichia coli*. Its antibacterial strength significantly increased with higher hinokitiol concentrations. Additionally, the hydrophobic properties of juniper mercaptan slowed the hydrogel membrane’s degradation, prolonging the antibacterial agent’s release. This sustained release mechanism enables the membrane to maintain an effective antimicrobial effect for at least 2 weeks, offering a promising antimicrobial treatment option. Chitin, known for its biocompatibility, biodegradability, non-toxicity, and antibacterial properties, can be transformed into CS, a polysaccharide widely used in biomedicine (Elizalde-Cárdenas et al., 2024). Carboxymethyl chitosan, a chemically modified version of CS, can be grafted with a photosensitive methacrylic acid group to create a novel polymer. This polymer quickly formed a 3D network architecture when exposed to blue light, making it suitable as a barrier membrane due to its biocompatible, degradable, and antibacterial characteristics (Xing et al., 2022).

**FIGURE 1:**
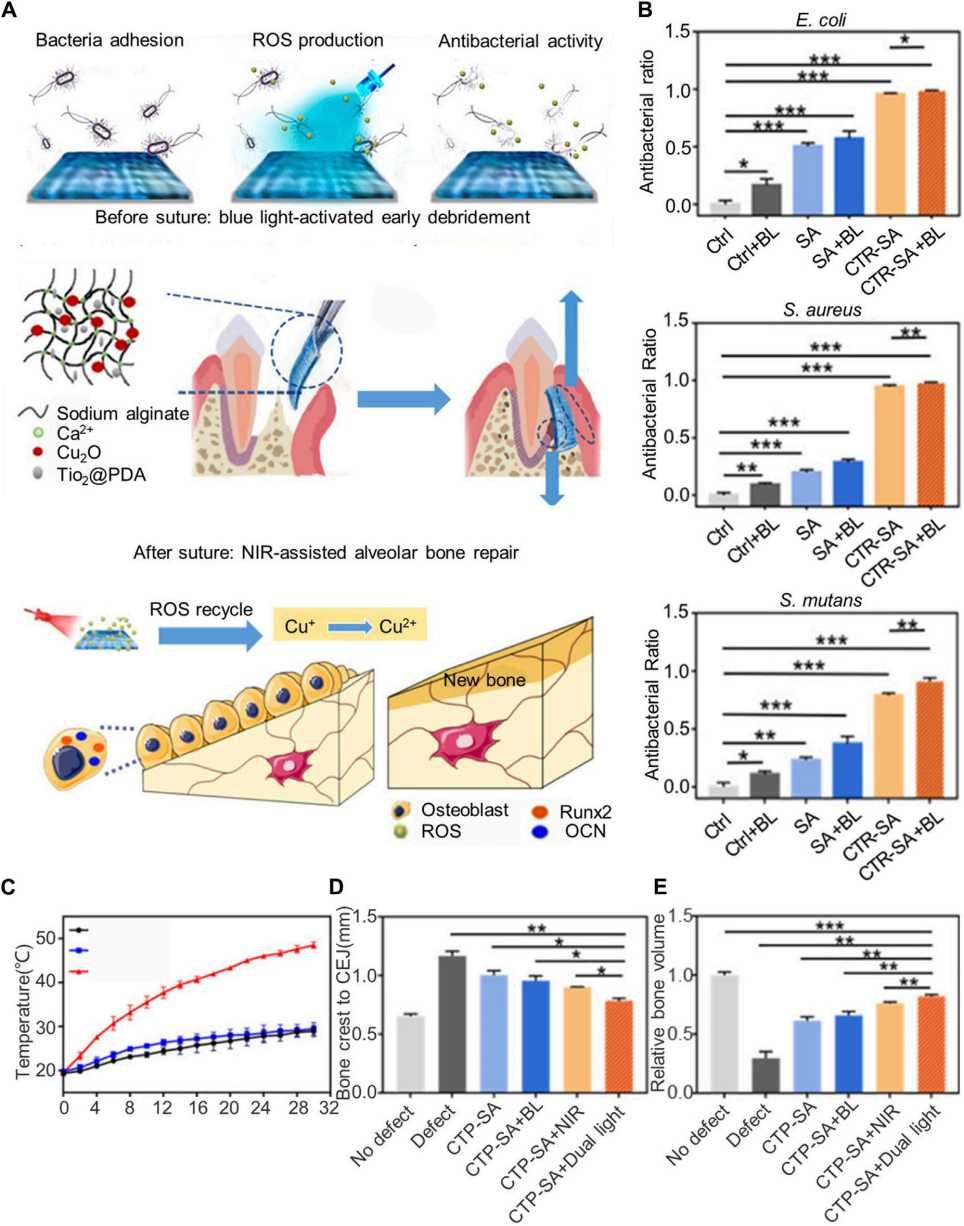
Dual-light-tunable sodium alginate hydrogel used as a barrier membrane for GTR with antibacterial/osteogenic mode switch properties. **(A)** Schematic illustration of the material composition, experimental procedure, and therapeutic mechanism of CTP-SA. **(B)** *In vitro* antibacterial effects of blue light irradiation on *Escherichia coli*, *Staphylococcus* aureus, and *Streptococcus mutans*. **(C)** Photothermal curves under NIR irradiation. **(D,E)** Evaluation of the effect of dual light-modulated hydrogels on osteogenesis *in vivo*. **(D)** Quantitative statistics of the distance from the bone crest to CEJ. **(E)** Relative bone volume. Reprinted with permission from Xu et al. (2020). Copyright (2020) American Chemical Society.

Metals and metal oxides serve as broad-spectrum, long-acting antimicrobials, killing bacteria through the dissolution of metal ions (Abo-Zeid and Williams, 2020)and the generation of reactive oxygen species (ROS), which oxidize biological macromolecules (Kannan et al., 2020). Xu et al. (2020) developed a polydopamine-modified titania nanoparticle and cuprous oxide-doped injectable sodium alginate hydrogel (TiO_2_@PDA), which exhibited peroxidase-like activity under blue light, generating ROS to eliminate bacteria effectively and support early debridement. The ROS generated by TiO_2_@PDA also transformed Cu^+^ into Cu^2+^, enhancing the growth and osteogenic differentiation of bone mesenchymal stem cells (BMSCs). Moreover, polydopamine granted the titania nanoparticles a photothermal effect, producing thermal energy under near-infrared light to further promote bone regeneration. The unique light-sensitive properties of dual-photoresponsive composite hydrogels enableed them to alter their structure or chemistry under varying light conditions, seamlessly transitioning between antimicrobial and osteogenic functions (Figure 1). This adaptability allows the material to meet the specific needs of the treatment phase. For instance, the composite hydrogels can release ROS at targeted light wavelengths for strong antimicrobial action, or adjust light conditions to promote bone tissue growth. Additionally, non-metallic photosensitizers like temoporfin can be incorporated into the barrier membrane, offering a photodynamic antibacterial effect under appropriate light exposure.

Growth factors play a crucial role in regenerative medicine by enhancing the repair and regeneration of damaged tissues. Incorporating growth factors into GBR/GTR membranes results in their sustained release in the target area, thus more effectively stimulating the differentiation and proliferation of osteoprogenitor cells or similar cells, which speeds up the tissue regeneration process (Mirzaeei et al., 2022). Consequently, hydrogel bio-barrier membranes serve as effective carriers of growth factors, like bone morphogenetic proteins, to boost tissue regeneration efficiency (He et al., 2021). Considering the tendency of implants to displace and the frequent compression of periodontal tissues by external forces leading to suboptimal regeneration, recent research has focused on enhancing the adhesion capabilities of barrier membranes to resist external stimuli. Li et al. (2023) introduced a dual-layer adhesive barrier membrane combining a collagen membrane with a polyacrylamide/polydopamine hydrogel. The dopamine’s catechol group chemically bonded to the bone surface, creating a strong adhesive effect. In tests, this membrane demonstrated high adhesive strength, exceeding 90 kPa, ensuring a robust bond to the bone surface. When used with bone graft material, it effectively retained nearly all the grafts, in contrast to the minimal retention observed with traditional collagen membranes. This indicated its superior fixation and bone repair capabilities, with significant alveolar bone regeneration observed 25 weeks post-transplantation.

Advances in materials science have significantly propelled the regeneration of periodontal and bone tissues. The evolution from early non-absorbable membranes to later absorbable ones, and now to contemporary multifunctional bio-barrier membranes that combine the advantages of both, has markedly advanced regenerative medicine technology. Hydrogel bio-barrier membranes have demonstrated promising clinical application potential in both cell and animal models, promoting cell adhesion and proliferation and providing an ideal microenvironment for tissue regeneration. Nonetheless, further research into the precise biological mechanisms of these hydrogel materials in clinical settings is necessary to fully optimize their efficacy.

## 3 Hydrogels for periodontal tissue engineering

### 3.1 Hydrogels as scaffolds

Scaffolds play a pivotal role in tissue engineering, aiming to replicate the composition of the ECM by providing 3D structural support and guidance for both exogenous and endogenous cells (Yamada et al., 2022). Hydrogels are particularly valued in this field for their unique 3D network structure and high water content, resembling the natural ECM closely (El-Sherbiny and Yacoub, 2013). To enhance tissue regeneration, numerous innovative hydrogel scaffolds have been developed to improve the interaction between target tissue cells and scaffold materials.

When designing hydrogels to serve as scaffolds for periodontal regeneration, it is crucial to consider the material, structure, and functionalization techniques to ensure they meet specific design requirements (Gorriz et al., 2015; Naahidi et al., 2017). These include the following: (a) biocompatibility: It is essential that the hydrogel scaffolds are compatible with the target tissue. After their synthesis, any remaining monomers, initiators, or other components not involved in the reaction could harm the tissue. Therefore, careful selection of materials and synthesis processes, followed by thorough purification, is necessary to minimize any potential adverse effects; (b) biodegradability: Hydrogel scaffolds should degrade and resorb in a controlled manner *in vivo*; (c) porosity: A key property of hydrogel scaffolds is their porosity (Chen et al., 2018), which allows nutrients and oxygen to reach cells and waste products from metabolism to be expelled. Suitable, interconnected pores will facilitate cell growth into the scaffold, and uniformly distributed cells will aid in tissue regeneration. Pore characteristics, including size, shape, and volume distribution, are crucial factors affecting a scaffold’s porosity. These characteristics influence cell penetration distance within the scaffold. Mechanical strength is essential for the scaffold to integrate into the damaged area and support weight. Additionally, the hydrogel scaffold’s surface properties are vital for influencing cell adhesion and proliferation by interacting with surrounding tissue. Through comprehensive consideration and optimization of these characteristics, hydrogel scaffolds can provide an ideal 3D microenvironment that significantly promotes the repair of periodontal tissue. Pan et al. (2020) developed an injectable hydrogel scaffold containing nano-hydroxyapatite based on polysaccharides. This scaffold showcased excellent microstructure and mechanical properties, along with a porous structure that supports the migration of endogenous stem cells. After implantation in a rat model of alveolar bone defects for 4 weeks, the scaffold completely degraded, leading to substantial alveolar bone regeneration without any significant inflammatory reactions. Additionally, incorporating nano-hydroxyapatite into porous hydrogel scaffolds made from polyvinyl alcohol and alginate (Bahrami et al., 2019) could mitigate the scaffolds’ mechanical weaknesses, increasing their compressive strength to approximately 3.51 MPa. These alginate/polyvinyl alcohol scaffolds with hydroxyapatite nanoparticles also exhibited superior cell attachment and promoted growth and differentiation of PDLSCs into osteoblasts, thus creating an optimal environment for periodontal tissue repair.

In tissue engineering, selecting and using the right scaffold materials is crucial. Scaffolds provide the necessary 3D structure for cell adhesion and growth but often lack the intrinsic activity to stimulate cell proliferation and division. To enhance scaffold functionality, researchers frequently incorporate growth factors or other bioactive molecules. Incorporating growth factors into hydrogel scaffolds enables localized, controlled release of specific active ingredients, delivering vital bioactive signals at crucial tissue regeneration stages, fostering cellular responses, and guiding tissue repair and regeneration. He et al. (2019) developed high-stiffness transglutaminase gels enriched with interleukin-4 and/or stromal cell-derived factor-1α (SDF-1α) as scaffolds, which demonstrated that interleukin-4 could induce macrophages to adopt an anti-inflammatory M2 phenotype, promoting osteogenic development of BMSCs *in vitro*. Additionally, SDF-1α consistently enhanced the recruitment of BMSCs, resulting in effective bone regeneration. In another study, Momose et al. (2016) achieved alveolar bone reconstruction using a collagen hydrogel scaffold loaded with fibroblast growth factor-2 in a beagle dog model of a class II bifurcation deficit, without any abnormal healing. Similarly, collagen hydrogel scaffolds combined with bone morphogenetic protein-2 (BMP-2) have been shown to induce effective periodontal repair (Kato et al., 2015). Tan et al. (2019) encapsulated bioactive components in supramolecular hydrogels to promote alveolar bone reconstruction, co-assembling the biocompatible hydrogel agent Nap-Phe-Phe-Tyr-OH (NapFFY) with SDF-1 and BMP-2. Their findings indicated that these bioactive substances could be synchronously and continuously released, significantly enhancing BMSC recruitment, osteogenic differentiation, and alveolar bone regeneration. Rats treated with the SDF-1/BMP-2/NapFFY hydrogel for 8 weeks exhibited a significantly higher bone regeneration rate (56.7% bone volume fraction) compared with controls. The results of the study revealed that two bioactive compounds could be synchronously and continuously released from a hydrogel, effectively enhancing the recruitment of BMSCs, osteogenic differentiation, and the regeneration of alveolar bone. Rats with bone defects, treated with the SDF-1/BMP-2/NapFFY hydrogel for 8 weeks, experienced significantly improved bone regeneration, with a bone volume fraction of 56.7%, compared to the control group.

**FIGURE 2:**
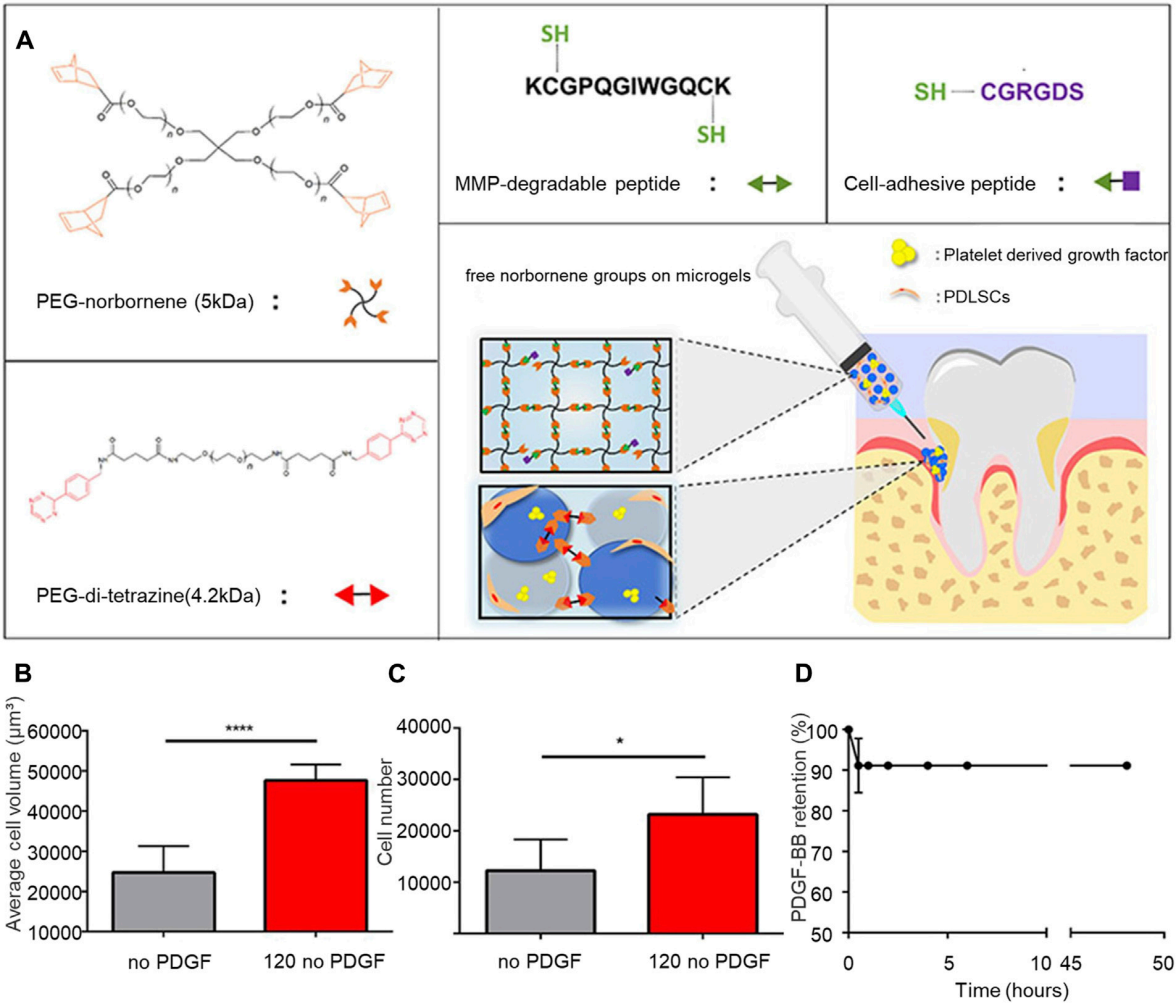
TzMAP hydrogel for periodontal regeneration. **(A)** Overview of hydrogel materials and use. **(B)** Average cell volume of PDLSCs at 5 days **(C)** Number of cells per 5 μL of hydrogel observed at 50 days. **(D)** Changes in the content of PDGF-BB in the TzMAP hydrogel over time. Reprinted with permission from Isaac et al. (2019). Copyright (2019) American Chemical Society.

During periodontal regeneration, the hydrogel scaffold acts as carriers for stem cell transplantation. The differentiation of these stem cells can be further promoted by altering the hydrogel scaffold, thereby impacting the regeneration of periodontal tissue. Utilizing a 4-amino-DL-phenylalanine (Phe-NH) dynamic acyl hydrazone crosslinker, Yang et al. (2021) developed an injectable chitin hydrogel system. This method encapsulated rat bone marrow-derived stem cells (rMSCs) within the hydrogel. After 7 days, the rMSCs were evenly distributed throughout the hydrogel, with over 95% cell survival. The hydrogel was observed to boost the differentiation of rMSCs, as indicated by a marked increase in marker expression. For periodontal tissue regeneration, scaffolds, external stem cells, and growth factors can be used in combination. For periodontal tissue regeneration, scaffolds, external stem cells, and growth factors can be used in combination. Isaac et al. (2019) created polyethylene glycol hydrogel microparticles using immersion electrospray, then formed microporous annealed granular hydrogels (TzMAP hydrogels) by linking norbornene-containing polyethylene glycol hydrogel microspheres through tetrazine click chemistry. Integrating PDLSCs during annealing showed that cell viability after 24 h showed that the survival rate of PDLSCs was approximately 87% ± 5%. The incorporation of PDLSCs and platelet-derived growth factor-BB (PDGF-BB) into TzMAP resulted in increased cell proliferation and migration, foundational for periodontal tissue regeneration (Figure 2). Ammar et al. (2018) mixed platelet concentrates into thermosensitive CS/β-glycerophosphate hydrogels, demonstrating that TGF-β1 and PDGF-BB could be continuously released for up to 2 weeks, thereby sustaining the viability of encapsulated PDLSCs for 7 days and promoting periodontal regeneration.

Furthermore, functionally graded scaffolds have been developed for the regeneration of multiple tissues, acknowledging that periodontal regeneration involves complex tissues and structures. Sowmya et al. (2017) introduced a three-layer scaffold system for periodontal tissue regeneration that includes a layer for cementum regeneration using CS/poly (lactic-co-glycolic acid)/nanobioactive glass-ceramic/cementum protein 1, a CS/PLGA/fibroblast growth factor-2 layer for PDL regeneration, and a CS/PLGA/nano-sized bioactive glass/PRP layer for bone regeneration. This system achieved simultaneous regeneration of cementum, PDL, and alveolar bone.

Although various hydrogels exhibit promising effects in periodontal restoration and are prepared using advanced methods, commercial options remain limited. This scarcity stems from the complex manufacturing processes of composite hydrogels, which often require specialized formulations and equipment, leading to longer preparation times and significantly higher costs compared to simpler hydrogel products. Additionally, the impact of different structural and physicochemical characteristics on periodontal restoration is not well-understood, compounded by numerous confounding factors. These challenges considerably hinder the clinical application of hydrogels with complex structures.

### 3.2 Hydrogels as carriers for cell transplantation

Stem cells, known for their self-replication capability and versatility in developing into various functional cell types, present a significant potential in cell repair, regenerative medicine, and pharmacology (Li B. et al., 2021; Bartold and Ivanovski, 2022). This potential is largely due to their underdifferentiated state. Research has extensively examined stem cells’ ability to regenerate periodontal tissue, identifying two main categories: odontogenic and non-odontogenic stem cells. Odontogenic stem cells include dental pulp stem cells, stem cells from human exfoliated deciduous teeth, PDLSCs, apical papilla stem cells, and gingival mesenchymal stem cells (GMSCs). By contrast, non-odontogenic stem cells encompass BMSCs, adipose-derived stromal cells, embryonic stem cells, and induced pluripotent stem cells. A wealth of animal studies and clinical reports supports the effectiveness of stem cell therapy in addressing periodontal tissue defects (Vandana et al., 2017; Kandalam et al., 2021; Fonticoli et al., 2022; Iwasaki et al., 2022; Shaikh et al., 2022). For successful tissue regeneration, stem cells must efficiently reach and function within the lesion, making cell transplantation essential. However, the approach faces challenges such as low survival rates and difficulty in cell engraftment, underscoring the need for better cell delivery mechanisms. Carrier materials for periodontal regenerative stem cells should support cell differentiation and growth (Ding et al., 2021), with specific requirements for optimal performance: appropriate pore size and porosity for physiological functions, biological strength, biocompatibility, tissue adhesion, controlled degradation with non-toxic by-products, and sterilization compatibility. Hydrogels, known for enabling metabolite, oxygen, and nutrient diffusion (Li et al., 2018), have emerged as promising stem cell carriers. They can be tailored to specific needs by adjusting their composition, properties, and fabrication methods, offering a versatile platform for cell delivery.

One advantage of hydrogels is their ability to be directly injected into the lesion area through minimally invasive procedures. A research team, led by Kandalam et al. (2021), successfully encapsulated pre-differentiated GMSCs in a self-assembled hydrogel known as PuraMatrix™. The GMSCs, encapsulated with 0.5% PuraMatrix, exhibited remarkable adhesion and proliferation rates. Earlier discussions in this review highlighted that DN hydrogels possess superior mechanical properties compared to single hydrogels, enhancing their utility in cellular delivery. Choi et al. (2019) developed DN hydrogels by combining gellan gum, a natural polysaccharide, with poloxamer-heparin. These hydrogels, referred to as PoH/GG DNH, were used to culture rabbit BMSCs. The results indicated that PoH/GG DNH not only served as an effective cell culture medium but also significantly improved osteogenic differentiation and cell proliferation in both lab and animal studies. Ding et al. (2021) employed the Knoevenagel condensation process to create a novel hydrogel by gelling dextran, functionalized with benzaldehyde and cyanoacetate groups. This was followed by photocrosslinking 4-arm acrylated polyethylene glycol to form a secondary, enhanced network within the hydrogel. This composite hydrogel demonstrated exceptional injectability, self-healing properties, and the ability to protect cells from mechanical damage during injection. Additionally, the second network exhibited remarkable stability, acting as a scaffold post-transplantation, while the first network rapidly hydrolyzed, facilitating cell proliferation and diffusion.

The lack of a microporous structure in conventional hydrogels often hinders cell spreading and proliferation. While increasing the porosity of the hydrogel by reducing polymer concentration is a possible adjustment, it may compromise the hydrogel’s stability. A viable solution is the development of microporous hydrogels, which enhance both host and encapsulated cell motility. A previous study introduced gelatin and GelMA-based fast-curing microporous hydrogels (Edwards et al., 2022). These hydrogels, which set within 2.5 min of injection through photopolymerization and enzymatic cross-linking, allowed for uniform cell distribution and significant cell spreading and proliferation within a week. Additionally, these hydrogels could transport hMSCs primed with interferon-gamma, boosting the release of anti-inflammatory agents such as prostaglandin E2 and interleukin-6. Hence, these hydrogels present a promising cell delivery mechanism. Hydrogel particles (HMPs) offer another protective means for cell transplantation. Consisting of biopolymer-made, tiny 3D network structures, HMPs protect cells from shear stresses during transplantation, thus preserving cell viability (Blaeser et al., 2016; Chen et al., 2017; Mealy et al., 2018). For instance, a capillary microfluidic device was employed by Zhao et al. (2016) to produce GelMA HMPs encapsulating BMSCs. Over 4 weeks, these BMSC/GelMA cultures showed a significant increase in DNA content, indicating effective cell migration and viability. Further, Teng et al. (2022) demonstrated that GelMA hydrogel microspheres could act as carriers for rat BMSCs without compromising cell survival rate of almost 99%. Following implantation in a rat bone defect model, CT scans revealed significant bone regeneration at the defect site after 8 weeks.

Hydrogels can be precisely engineered for controlled cell distribution and tissue structure formation using self-assembly or 3D printing techniques. A bioprinting approach utilized injectable composite hydrogels composed of GelMA and polyethylene glycol dimethacrylate to encapsulate PDLSCs (Ma et al., 2017). The addition of polyethylene glycol enhanced droplet management. Hydrogels containing PDLSCs showed increased bone growth in rat alveolar bone defects compared to hydrogels without cells, as evidenced by *in vivo* experiments.

While hydrogels have shown promise as carriers for stem cell transplantation, challenges remain. Issues such as cell death during implantation, low cell retention post-transplantation, difficult long-term cell survival, and inadequate structural support significantly impact the effectiveness of periodontal regeneration therapy. Currently, there is no ideal hydrogel cell carrier that addresses these concerns.

### 3.3 Hydrogels as drug delivery systems

Over the last decade, studies have shown that various medications and growth factors can promote periodontal tissue regeneration (Liang et al., 2020), as detailed in Table 1. However, the limited application of these bioactive substances is often due to their short half-lives and rapid degradation rates. To address this issue, researchers have developed strategies to mitigate the drug’s adverse effects and extend its duration of action (Onaciu et al., 2019; Pushpamalar et al., 2021). One popular method involves using hydrogels for drug delivery. Due to their unique physical properties, hydrogels are ideal for this purpose, allowing for sustained drug release. Drugs can be encapsulated within hydrogels, integrated into their structures, or adsorbed on their surfaces. Various factors influence drug release from hydrogels, including the hydrogel’s composition, shape, manufacturing method, external conditions, and the interaction between the hydrogel and the bioactive substance (Zięba et al., 2020). By fine-tuning these factors, the rate and duration of drug release can be precisely controlled. The ideal hydrogel drug carrier should exhibit several biophysical and chemical characteristics. It must be biocompatible, not eliciting adverse reactions or immune rejection, and should degrade gradually in the body. The degradation byproducts should be non-toxic and non-inflammatory, with a degradation rate that matches the drug release, ensuring the drug’s efficacy for the required duration. Furthermore, the hydrogel must adhere securely to periodontal tissue to prevent displacement during treatment or daily activities.

**TABLE 1:** Summary of bioactive molecules for periodontal regeneration.

Bioactive molecules	Characteristics	Functions	Composition of hydrogels	Applications	References
Drugs					
Statins: Simvastatin; Atorvastatin	Drugs used to lower blood levels of low-density lipoprotein cholesterol and triglycerides	Inhibits periodontal inflammation primarily through ERK, MAPK, PI3-Akt, and NF-κB pathways	Pyrophosphorolated pluronic F127	Simvastatin loaded in a thermosensitive hydrogel was effective in reducing inflammation and preserving periodontal bones when evaluated in a rat periodontitis model	Chen et al. (2021)
Aspirin	Analgesic and antipyretic	Activates telomerase reverse transcriptase or inhibits tumor necrosis factor-α and interferon-γ pathways to enhance the osteogenic potential of MSCs	CS, β-Sodium Glycerophosphate and gelatin	Promoted PDLSC proliferation and osteogenic differentiation and improved *in situ* regrowth of new bones in a mouse model with cranial deficiency	Xu et al. (2019), Zhang et al. (2019, 2022)
Metformin	Widely used to treat type II diabetes	Promotes osteogenic differentiation and bone formation through AMP kinase activation. Regulates the production of NF-κB	Oxidized dextran and phenylboronic acid-functionalized poly (ethylene imine)	Good bone mass recovery when applied to a diabetic rat periodontitis model	Bahrambeigi et al. (2019), Zhao et al. (2022)
		Ligand, encourages the expression of osteogenic genes and ALP, reduces the number of osteoclasts, and inhibits osteoclast activity.			
Growth factors					
Platelet-derived growth factor (PDGF)	There are 4 PDGF genes that together form 5 dimeric isoforms, namely, PDGF-AA, PDGF-BB, PDGF-CC, PDGF-DD, and PDGF-AB	Promotes angiogenesis as well as proliferation and differentiation of osteoblasts, which facilitates bone formation	Thermosensitive poly (D, L-lactide-co-glycolide)-poly (ethylene glycol)-poly (D, L-lactide-co-glycolide) triblock copolymers	Promotes PDLSC proliferation and osteogenic differentiation and enhances PDLSC-mediated alveolar bone regeneration	Ammar et al. (2018), Pan et al. (2019)
Stromal cell–derived factor-1 (SDF-1)	Is a small-molecule cytokine that belongs to the chemokine protein family	Recruits stem cells, promotes cell proliferation, and activates cellular osteogenic capacity	Polyethylene glycol diacrylate	Promotes the proliferation, migration, and osteogenic differentiation of PDLSCs	Liu et al. (2021, 2022)
Bone morphogenetic protein family (BMP)	Subfamily of the TGF-β superfamily	Induces BMSC differentiation into bones, blood vessels, ligaments, and cartilage	Nap-Phe-Phe-Tyr-OH (NapFFY) hydrogel	BMP-2 has mainly osteogenic properties but may cause tooth straightening and root resorption	Chiu et al. (2013), Kato et al. (2015), Chien et al. (2018), Tan et al. (2019), Zang et al. (2019)
Plant extracts					
Berberine	Berberine is an alkaloid that can be found in many different plants. It has been used to treat several conditions, including cancer, type 2 diabetes, irritable bowel syndrome, hypertension, hyperlipidemia, and severe diarrhea	Anti-inflammatory and osteogenic effects are exerted through the PI3K/AKT signaling pathway	Sodium alginate, CS and β-glycerophosphate	Promotes alveolar bone regeneration to a certain extent	Wang et al. (2023)

**FIGURE 3:**
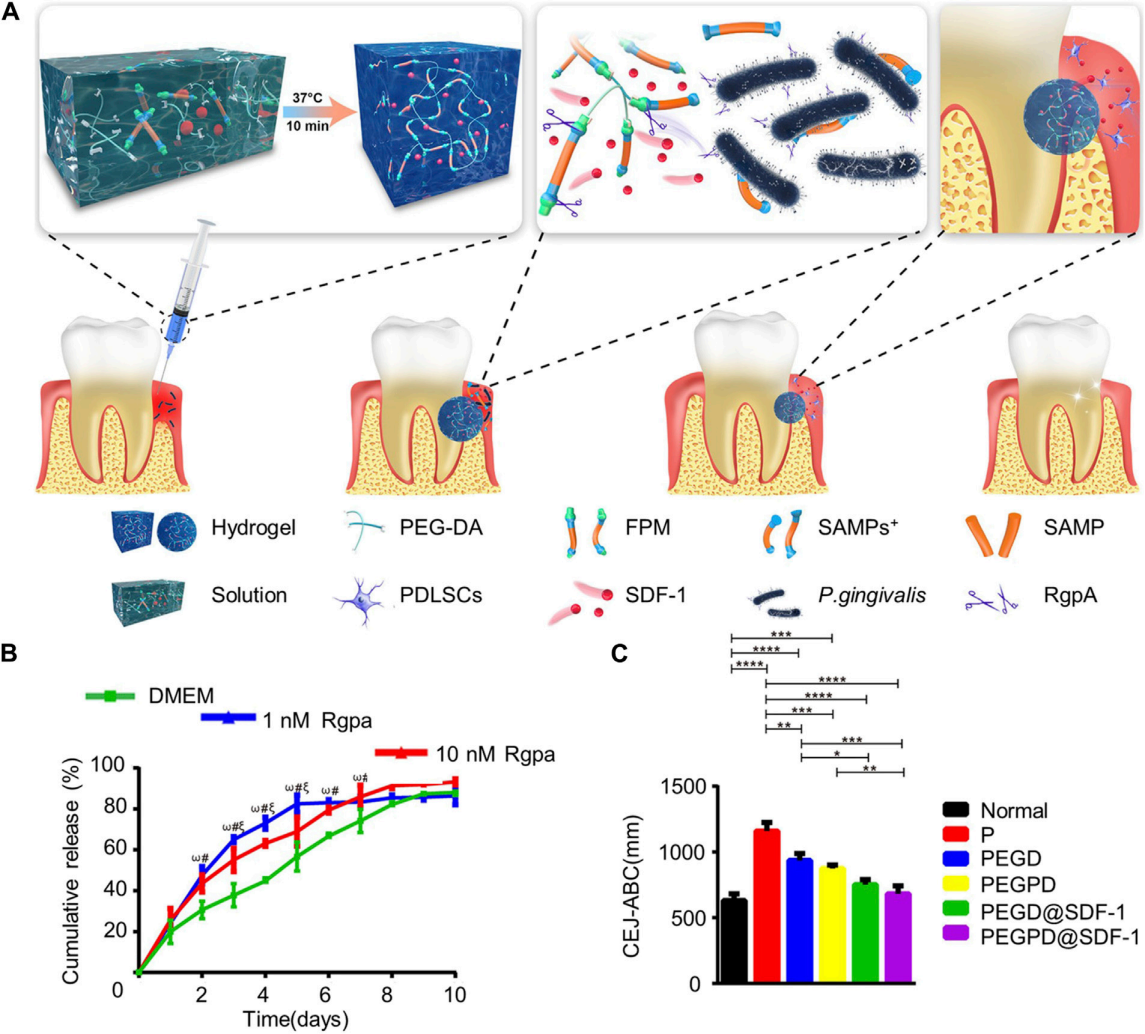
SDF-1-loaded gingival protease-reactive thermothermal hydrogel promotes periodontal regeneration. **(A)** Schematic representation of the hydrogelmaterial composition and processing. **(B)** Release curves of SDF-1 from the hydrogel. **(C)** Distance between CEJ and ABC *in vivo* model 4 weeks after treatment. Reprinted with permission from Liu et al. (2021). Copyright (2021) American Chemical Society.

In recent years, there has been growing interest in the development of stimulus-responsive or “smart” hydrogels (Huang et al., 2020). These hydrogels can change shape or phase in response to environmental stimuli, such as temperature changes, facilitating drug release through swelling-shrinking actions or gel-sol transitions. Thermosensitive hydrogels, for example, are in liquid form at low temperatures but gelate at higher temperatures, a property crucial for effective drug delivery. Wang et al. (2023) developed a berberine heat-sensitive hydrogel that gels rapidly at body temperature (37°C) within approximately 3 min, demonstrating efficacy in treating periodontal inflammatory bone conditions by modulating the PI3K/AKT signaling pathway and offering anti-inflammatory and osteogenic benefits. Xu et al. (2019) created an injectable thermosensitive hydrogel that continuously released aspirin and erythropoietin, facilitating the healing of inflammation and alveolar bone repair. Aspirin and erythropoietin released 86.6% and 69.4% of their doses, respectively, within the first 3 days of the drug release trial, with aspirin’s faster release rate aiding in the early stages of periodontitis treatment where controlling inflammation quickly is vital. The thermosensitive hydrogel developed by Liu et al. (2022) useed polyethylene glycol diacrylate as a basal scaffold and imparted excess ROS–scavenging effects to the hydrogel and oxidative stress–reducing effected to cells by adding the reducing sugar alcohol dithiothreitol, thereby protecting periodontal cells from inflammatory damage. The inclusion of SDF-1α increased the hydrogel’s biological activity, promoting cell proliferation and differentiation by activating the Wnt/β-catenin signaling pathway, essential for alveolar bone regeneration. They also introduced a new functional peptide module responsive to arginine protease secreted by *Porphyromonas gingivalis*, creating a smart gingival protease-reactive hydrogel (Liu et al., 2021). This hydrogel generated short antimicrobial peptides activated by arginine protease, inhibiting the growth of *P. gingivalis*. Under the action of different concentrations of gingival protease R1 protein, the release rate of SDF-1 in hydrogels increased with the increase of protease concentration, because protease would cut off the functional peptide module in the hydrogel and destroy the structure of the hydrogel, which was conducive to the antibacterial function of antimicrobial peptides and the function of SDF-1 to recruit host stem cells and promote osteogenesis, as illustrated in Figure 3.

Inflammation in periodontal tissues leads to an increase in ROS levels, prompting the development of ROS-reactive materials for periodontal disease treatment. The hydrogel system designed by Zhao et al. (2022) used the presence of ROS to initiate drug release. This system, made from oxidized dextran and phenylboronic acid-functionalized polyethylenimine, enhances the efficiency of doxycycline and metformin delivery through B-N ligands. It achieves localized drug release in response to ROS, boosting the combined therapeutic benefits of doxycycline’s antimicrobial action and metformin’s bone regeneration promotion. Additionally, the alteration of pH within the periodontal pocket during disease progression offers a chance to develop pH-sensitive hydrogels that respond to this change, aiding in periodontal tissue regeneration. pH-sensitive hydrogels, which are protonation polymers containing acidic or basic groups, change their volume in response to pH alterations, either releasing or absorbing protons. Yu et al. (2016) developed a pH-responsive hydrogel containing anti-glycation agents that slowed the progression of periodontitis and promoted recovery. Beyond environmentally sensitive hydrogels, there are also stimulus-responsive hydrogels and multisensitive hydrogels that offer combined advantages for drug delivery mechanisms, showing significant potential for future applications (Lin et al., 2020; Hsia et al., 2024).

In addition to stimuli-responsive hydrogels, the structure of hydrogels formed by a dual dynamic network can be tuned to manage the controlled release of drugs effectively (Bertsch et al., 2022). The innovative injectable and self-healing hydrogel developed by Guo et al. (2021) is aimed at facilitating periodontal regeneration. This hydrogel, synthesized from aldehyde-modified hyaluronic acid (HA-CHO) and ethylene glycol chitosan (GC), formed a dual dynamic network through the dynamic Schiff base bonds between the -CHO group in HA-CHO and the -NH_2_ group in GC, alongside dynamically oriented bonds between the COO- group and Fe^3+^ ions in HA-CHO. Owing to its unique structure, the hydrogel exhibited robust self-healing capabilities, enabling it to swiftly regain its mechanical properties even after being subjected to high strain (e.g., 500%) and return to its original state under low strain (e.g., 1%). Moreover, by adjusting the biodynamic bonds, the hydrogel’s drug release rate could be customized to meet specific therapeutic needs. In their study, the researchers demonstrated this capability by loading the hydrogel with ginsenoside Rg1, known for its anti-inflammatory properties, and enamel for its regeneration-promoting effects, under varying concentrations of FeCl_3_ to control the release duration. The results indicated the potential for precise control over drug release timing through the adjustment of biodynamic linkages in hydrogels. Significant periodontal regeneration in rats with periodontal defects showcased the hydrogel’s potential in promoting tissue regeneration, offering a novel solution for periodontitis management.

While *in situ* hydrogels offer a degree of local pharmaceutical storage, achieving sustained local delivery of hydrophilic small molecules remains a challenge. Microspheres, however, are recognized for their effective long-acting sustained-release property (Zhang et al., 2018). The introduction of microspheres into hydrogels introduces two physical barriers that the drug must overcome, enabling prolonged therapeutic effects. A research group designed a hydrogel system for the sequential release of basic fibroblast growth factor and transforming growth factor-beta 3 (Yu et al., 2022). In this system, transforming growth factor-beta 3 was encapsulated in CS microspheres before being co-loaded with basic fibroblast growth factor into CS hydrogels, facilitating a staggered release that aligns with different healing phases. Notably, the system demonstrated significant alveolar bone repair 12 weeks after being injected into an alveolar bone defect model. Wang B. et al. (2020) developed an innovative topical drug delivery system for periodontal applications by utilizing electrospraying to encapsulate two medications into PLGA microspheres within a thermoreversible polyisocyanate hydrogel. This method offers key advantages, including biosafety, injectability, and enhanced structural stability over time. By adjusting the load-to-mass ratio of acid-terminated PLGA, the release of the drug could be regulated. An adjustable and injectable topical drug delivery system was developed by incorporating two drugs into PLGA microspheres within a thermoreversible polyisocyanate hydrogel through electrospray. This technology offers biosafety, injectability, and long-term structural stability. Adjusting the load-to-mass ratio of acid-terminated PLGA allows for precise manipulation of drug release.

The precise spatial and temporal control of bioactive signals is essential for their anti-inflammatory and regenerative effects. Proper timing is critical in controlling inflammation, a necessary step for tissue regeneration, as is the timely administration of growth factors for the regeneration of various periodontal tissues. Despite the development of numerous hydrogel drug delivery systems, achieving exact control over the delivery of bioactive molecules for periodontal tissue regeneration remains a challenge.

## 4 Conclusion and perspectives

Hydrogels have significantly advanced periodontal regeneration therapy due to their excellent biocompatibility and structural resemblance to the ECM, allowing for their widespread use in this field. They serve various roles, including as GTR membranes, tissue engineering scaffolds and drug delivery systems, contributing to periodontal regeneration. However, despite promising experimental results, hydrogels have not yet achieved successful clinical outcomes in periodontal regeneration. The challenges in utilizing hydrogels for functional periodontal tissue regeneration are as follows:

Periodontal tissue regeneration is complex, involving multiple cell and tissue types with differing structures, compositions, and functions. This complexity makes it challenging for a single hydrogel to effectively mimic these characteristics. Current strategies often focus on regenerating a single tissue type, such as alveolar bone, without adequately addressing the regeneration of the PDL diverse fiber bundles. The natural regenerative capacity of periodontal tissue is limited, particularly in the presence of infection and inflammation. Enhancing regeneration often requires external interventions, such as cell transplantation or recruiting stem cells from surrounding tissues. For effective periodontitis treatment, the drug delivery system must precisely control the release timing and location of medications, transitioning from anti-inflammatory to tissue-regenerating agents. While hydrogels can provide controlled release in response to stimuli, they fall short in precisely regulating periodontal tissue regeneration. This limitation stems from an incomplete understanding of the regeneration and healing process and the limited efficacy of bioactive molecules in promoting regeneration. The mechanical strength of hydrogels is another limiting factor for their application in periodontal tissue engineering. Achieving a balance between mechanical strength and injectability is challenging, as enhancing mechanical properties often reduces injectability. Moreover, rapid degradation of hydrogels *in vivo*, influenced by factors such as the inflammatory microenvironment, differs from *in vitro* results and requires further research. Despite these obstacles, hydrogels remain a promising area of research for periodontal tissue regeneration. Ongoing development of hydrogel systems capable of repairing periodontal tissue offers hope for improved treatment outcomes and innovative solutions for periodontal patients in the future.
